# Resources and interventions to support psychological health and wellbeing in the pharmacy workforce: Analysis and use of a health worker ‘burnout’ toolkit^☆^

**DOI:** 10.1016/j.rcsop.2023.100359

**Published:** 2023-11-03

**Authors:** Katrina Mulherin, Jaden Brandt, Amy Hu, Pavithra Ravinatarajan

**Affiliations:** a Canadian Pharmacists Association - Workforce Wellness Task-Force, 851 Industrial Avenue, Mailbox M035, Ottawa, ON K1G 4L3, Canada; b Windpharm Consulting, Fredericton, NB, Canada; c College of Pharmacy, Rady Faculty of Health Sciences, University of Manitoba, Winnipeg, MB, Canada; d Holland Bloorview Kids Rehabilitation Hospital, Toronto, ON, Canada; e School of Pharmacy, University of Waterloo, Kitchener, ON, Canada

**Keywords:** Pharmacy, Burnout, Mental health, Workforce, Human resources, Workload, Occupational health, Moral distress

## Abstract

**Background:**

Pharmacists have experienced declines in psychological health and wellbeing post-pandemic. The phenomena of moral distress, disengagement and burnout are associated with workforce attrition, unfitness to practice and inferior quality of patient care. A working group of the Canadian Pharmacists Association (CPhA) was formed to identify resources and interventions (R&I) for occupational psychological health and wellbeing.

**Objective:**

To characterize R&I from an evidence-based national health worker ‘burnout’ Toolkit with potential to support the pharmacy workforce.

**Methods:**

All R&I included within a draft ‘burnout’ Toolkit from the Canadian Health Workforce Network (CHWN) were screened to determine relevancy and usefulness for the pharmacy workforce. R&I with higher grades were data-charted to capture information on topic and content delivery. Final R&I were determined through consensus meetings where ‘highly rated’ R&I were discussed and selected.

**Results:**

Of 140 original CHWN Toolkit R&I, 53 (37.8%) were of *potential* relevance or usefulness to improve well-being for *most* in the pharmacy workforce. Of those 53 R&I, 28 (20% of original) were final selections. The majority of R&I at each stage were focused on ‘preventing burnout’ and ‘promoting mental health’ (>60%) rather than ‘addressing burnout’, ‘supporting recovery’ or managing specific issues in the workplace (i.e. stigma, discrimination, bullying, hostility, workload). No R&I were specifically developed or studied within the pharmacy workforce.

**Conclusions:**

Health professions may benefit from the CHWN Toolkit and the knowledge translation activity described here. R&I relevant and useful to the pharmacy workforce generally require adaptation for dissemination and/or implementation. The set of final R&I form the basis for orchestrated plans to support the pharmacy workforce with respect to psychological health and wellbeing. There is a relative lack of R&I devoted to addressing and recovering from burnout and workload management issues.

## 1 Introduction

Pharmacists are experiencing declines in psychological health and wellbeing. Pre-pandemic evidence described occupational-related psychological phenomena of moral distress, disengagement, and burnout (the phenomena) these professionals experience.^1^^,^^2^ Post-pandemic evidence indicates the phenomena remain and have likely increased in frequency and magnitude.^3^^,^^4^ Workforce attrition and unfitness to practice are known consequences of the phenomena.^5^ Indeed, post-pandemic, there is evidence of accessibility issues for patients however, the degree attributable to the phenomena is unknown.^6^ There are gaps in quantitative workforce data across Canada describing inward and outward flow of pharmacists which contributes to a lack of understanding what impact on human resourcing in this sector.^7^ Recent Canadian indicators of the phenomena such as substance use and suicide rates if available, would be another informative measure relating to occupational psychological health and wellbeing of pharmacists.^8^ In Canada, the term ‘pharmacy professional’ encompasses pharmacists and pharmacy technicians. Significant research into the phenomena among pharmacy technicians is lacking.

International pharmacy graduates comprise 34% of pharmacists licensed to practice in Canada.^9^ This is greater than rates within other professions of occupational therapists, physicians, nurses and physiotherapists and indicates a correspondingly ethnically diverse profession highly vulnerable to abuse and microaggressions.^9^ The stresses underlying poor psychological health and wellbeing in pharmacy, and healthcare professionals more broadly, may be existential, societal, familial and/or occupational.^10^^,^^11^ These stresses intersect and compound for pharmacy professionals but especially those within minority groups in ways that are difficult to adequately describe and address by health workforce leaders, policy-makers, and healthcare administrators.^12^^,^^13^ There is research demonstrating that health professionals who are disengaged with their work provide their patients with inferior quality of care.^14^^,^^15^ Phenomena-associated workforce attrition has increasing potential to disrupt the accessibility of pharmacy and health services, especially in under-served populations (rural, remote and socially disadvantaged) thus placing patient health at risk.^16^, ^17^, ^18^

Pharmacy professionals are navigating growing practice complexity as a result of mounting drug shortages, widespread vaccination services, rising rates of patients with complex health needs, increased patient care responsibility (i.e. prescribing and laboratory testing), infection control measures, and workplace technology changes.^19^, ^20^, ^21^, ^22^, ^23^ Providing quality patient care in this dynamic milieu demands professionals expend progressively greater cognitive and physical effort.^24^ Community pharmacy practice is unique given the hybridisation of public/private healthcare, high public visibility and accessibility, physically remote location from other healthcare providers and volume of competing retail, technical, and professional demands.^25^ Community pharmacy services were largely maintained throughout the pandemic and pharmacists were increasingly called upon to solve issues routinely addressed by other members of the patient health care team.^26^^,^^27^ Community pharmacies' organisational structure is flat compared to most healthcare institutions, consisting of pharmacy staff (unregulated and regulated professionals) and a pharmacist manager. Additionally, chain pharmacies (i.e. non-independent) frequently operate under a further level of centralised corporate management, requiring responsive implementation of ‘top-down’ strategic initiatives developed outside of the immediate patient care environment.

Within this context, a national taskforce of pharmacists was convened by the Canadian Pharmacists Association (CPhA) in the summer of 2022. The taskforce's mandate was to address the phenomena on a national level. Its constitution was prompted by the results of the 2022 national pharmacy workforce survey that established a high rate of work-related burnout among pharmacists.^28^ Survey results suggest general consistencies with previous surveys investigating burnout, both pre and post-pandemic, among unique and varied samples of the international pharmacist workforce including but not limited to: Canada,^29^ the USA,^30^^,^^31^ U.K,^32^ Australia,^33^ Japan^34^ and Nigeria.^35^

The taskforce's immediate focus was to develop strategies to support pharmacists' occupational psychological health and wellbeing, with the understanding that significant delays in the identification, dissemination and implementation of resources and/or interventions (R&I) could negatively impact on retention of professionals within the workforce. To expedite efforts, a working group from the taskforce comprised of four pharmacists with diverse professional experience, background and geographical location was formed to identify R&I geared toward occupational psychological health and wellbeing. Formation of the working group (consisting of the authors) coincided with early draft publication in 2022 of the Canadian Health Workforce Network (CHWN) “Burnout Toolkit” (the CHWN Toolkit).^2^ CHWN is “a knowledge exchange network of researchers, decision-makers and other knowledge users with expertise in health workforce planning, policy and management”.^36^

CHWN produced the Toolkit catalogue of evidence-based psychological health and wellness R&I designed to support the Canadian healthcare workforce in dealing with burnout post-pandemic.^37^^,^^38^ CHWN organised the Toolkit's R&I in tables according to 3 major categories:

⋅Level of R&I: Systems/organisational/teams/individual

⋅Stage of burnout: Preventing/Addressing/Supporting Recovery

⋅Topic: Promoting Mental Health/Managing Workload/Handling Conflict, Bullying and Harassment/ Addressing or Confronting Discrimination/ Reducing Stigma and Facilitating Disclosure

While there has been some limited past publication on the adaptation, dissemination and/or implementation of R&I within pharmacy,^39^, ^40^, ^41^, ^42^ the taskforce determined strategies should preferentially build on this comprehensive, evidence-based catalogue of 140 R&I.^37^^,^^38^ The working group therefore undertook to translate knowledge within the CHWN Toolkit by extracting R&I, useful within the community pharmacy context, for mitigating the phenomena and for promoting occupational psychological health and wellbeing.

## 2 Methods

### 2.1 Overview

The working group's objective was to translate the knowledge encompassed within the CHWN Toolkit into community pharmacy-focused R&I recommendations that have potential benefit in both/either community pharmacy technician and/or pharmacist professions. The working group anticipated the results would be used to formulate an orchestrated national plan for occupational psychological health and wellness and so, developed an applied, practical and responsive approach to translating this research into a usable set of R&I.

The project commenced with a screening process where individual CHWN Toolkit R&I were graded on relevance and usefulness according to a pre-determined guide. The resulting set of R&I receiving high screening grades was recognised by the group as needing subsequent analysis to determine similarities between R&I and distribution across the three CHWN categories and to present a mix of R&I that could meet heterogeneous needs of the profession. To respond to this discovery, highly-graded R&I underwent subsequent data-charting to identify observable trends related to delivery and content (See Section: *Data-Charting* below). These R&I then proceeded to a second review process for distillation into a final set of R&I. R&I retained after the second review would have high potential in supporting occupational professional psychological health and wellness.

### 2.2 Protocol

Given the knowledge translation nature of this project, and the work having evolved in response to existing research, no protocol was selected or designed a priori*.* To the authors' knowledge, there are no reporting protocols established for the application of this type of research.^43^

### 2.3 Data collection

Data collection was manually derived directly from the CHWN Toolkit. Each of the 140 original R&I were accessed through electronic links provided within the CHWN Toolkit.

### 2.4 R&I grading guide

In preparation for screening the R&I, a grading guide was developed by one of the working group members (JB) and subsequently revised by the remaining three members. The final version of the grading guide (See Box 1) provided a standard approach to determining relevancy and usefulness of the R&I for the community pharmacy workforce via color-coding of the original CHWN tables. The grading guide did not consider feasibility of R&I however, it was agreed upon a priori that, R&I not judged adaptable or feasible for application to community pharmacy context and/or professionals would be maximally receive a yellow grade.

**Box 1:**
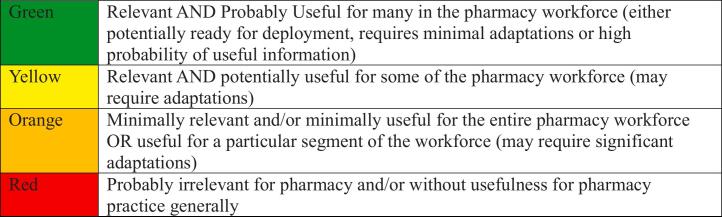
R&I grading guide.

### 2.5 Round 1: screening/grading

Each of the four working-group members was assigned one of the ‘levels’ to independently grade the individual R&I according to the grading guide **(Supplemental Appendix A)**. Grading of each member was corroborated by another member to ensure accuracy. Two virtual meetings were held to discuss grading decisions, resolve discrepancies, and achieve grading consensus within the group of four.

### 2.6 Round 2: data-charting

Data-charting was conducted in Microsoft Excel to collect and categorize information on various elements for each R&I with a green or yellow grade. In addition to the three original CHWN categories (level/stage of burnout/topic), an additional 5 variables (audience/medium/intervention type/interaction type/anonymity) were determined as important for analysis and development of an orchestrated national plan. (See Table 1 for detail and associated rationale**)**.

**Table 1:** Information collected from R&I in health worker burnout toolkit.

Round 2 - Categorisation Originally Conducted by CHWN			
Data Element	Response Variables	Comments on ‘Response Variable Distinction’	Rationale
Level	i) Systems ii) Organisational iii) Team iv) Individual	The organisational level may not apply to community pharmacies or primary care environments that do not operate within a larger institutional or corporate umbrella therefore, this level (depending on the R&I) may be perceived to fit either within the lower teams or upper systems level	Original category and data of the CHWN Health Worker Burnout Toolkit
Stage of ‘Burnout’	i) Preventing ii) Addressing iii)Supporting Recovery	*Preventing* – Measures taken to reduce the incidence of ‘burnout’ *Addressing* – Measures taken to manage ongoing symptoms and markers of ‘burnout’ *Supporting Recovery* – Measures taken to improve re-integration back into the workforce or improve mental health back to pre-burnout levels	Original category and data of the CHWN Health Worker Burnout Toolkit
Topic	i) Promoting Mental Health ii) Managing Workload iii) Handling Conflict, Bullying & Harassment iv) Addressing / Confronting Discrimination v) Reducing Stigma & Facilitating Disclosure	*Promoting Mental Health* – Focused on mental health proactively or reactively and for individuals with and without mental illness *Managing Workload* –Focused on improving workload by creating time or workplace efficiencies to align realistic work more adequately with professionals' capacity to perform optimally. *Handling Conflict, Bullying & Harassment –*Addressing individual person-to-person or group psychological, physical, or verbal conflict or abuse *Addressing/Confronting Discrimination –* Addressing bigotry or the targeting of individuals or groups based on appearance, lifestyle or immutable characteristics (i.e. race, biological sex, gender identity etc.) *Reducing Stigma & Facilitating Disclosure –* R&I focused on creating inclusive safe-spaces for mental health or sensitive identity disclosures.	Original category and data of the CHWN Health Worker Burnout Toolkit


Round 2– Additional Data-Charting on Green and Yellow Graded R&I Conducted by the Working Group			
Data Element	Response Variables	Comments on ‘Response Variable Distinction’	Rationale
Audience	i) Pharmacy ii) Healthcare iii) Occupations at risk iv) Societal	‘Pharmacy’ is nested within ‘healthcare’ which is nested within ‘occupations at risk’ which is nested within ‘societal’	To understand who the R&I is designed for
Medium	i) Electronic ii) Face to Face iii) Telephone iv) Virtual v) Other	*Electronic*: R&I that is accessed via the internet. *Virtual*: R&I that uses human to human interface via a virtual (video and audio) meeting platform	To understand how the R&I is accessed
R&I Type	i) Interactive ii) Passive	*Interactive*: R&I that requires the participant to act or engage in conversation or wellness activity *Passive*: Information that is presented for consumption in an informational way (print, video, audio) that requires the audience member's attention to gain knowledge, skill or attitudes	To understand the degree of effort and planning required on the part of participant(s) and/or providers to implement the resource/intervention. Psychological effectiveness may be higher for interactive interventions.
Interaction Type	i) Facilitated Group ii) One on one iii) Chatbot iv) Multiple offerings v) Print vi) Audio vii) Video	*Multiple offerings* indicate a mixed approach to the R&I such as readings as well as 1:1 opportunity. Some diverse platforms include multiple approaches to the programming	To understand the format in which the intervention/resource is engaged with. R&I could have been charted as using multiple formats.
Anonymized R&I	i) Anonymous ii) Not Anonymous	This categorisation may be relevant for professionals experiencing (actual or perceived) stigma	To understand the frequency with which certain types of R&I provided anonymity. Evidence shows pharmacists experience stigma as a barrier to accessing psychological health interventions so anonymity is relevant

### 2.7 Round 3: final selection

All four working-group members re-reviewed the most highly rated R&I (green and yellow ratings), along with their corresponding data-charted results **(Supplemental Appendix B**). For each of the ‘levels’ ranging from ‘systemic’ to ‘individual’, final R&I were selected via a consensus process over 2 virtual meetings. The working group originally aimed for selecting approximately 5 R&I for each ‘level’ (twenty total) by looking primarily at the highest rated (green) R&I. This number allowed for a manageable volume of R&I for recommendation but also compelled group members to make definitive decisions on the value of each R&I. However, through the discussions, an additional 8 R&I were retained due to their similar or complementary nature to other selected R&I and inferred need for detailed comparison prior to inclusion in an orchestrated national plan.

## 3 Results

**Fig. 1:**
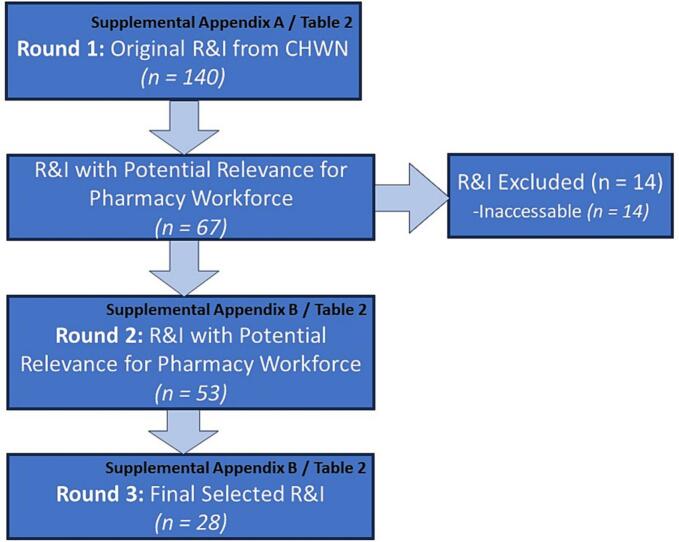
Screening and selection results.

Of the original 140 R&I (30 system, 50 organisation, 33 team and 27 individual), a total of 37 (26.4%) overall were graded as relevant and *probably* useful for *many* in the pharmacy workforce and 30 (21.4%) were graded as relevant and *potentially* useful for *some* in the pharmacy workforce. Of these 67 R&I rated high enough to explore further, 53 (79%) were sufficiently accessible (locatable and no associated cost) for further examination and data-charting. Frequencies of other data-charted variables for the yellow and green graded R&I that correspond to the variables in Table 1 of the methods are presented in Table 2 and separated according to each round depicted in Fig. 1**.**

**Table 2:** Observed frequencies of R&I characteristics.

Data Element	Response Variables	*Frequency (%) –* *Original Round 1 CHWN R&I* *(n = 140)*	*Frequency (%) –* *Round 2 R&I Selection*⁎ *(n = 53)*	*Frequency (%) –* *Final R&I Selection* *(n = 28)*
Level	i) Systemic	30 (21.4%)	12 (22.6%)	9 (32.1%)
	ii) Organisational	50 (35.7%)	10 (18.9%)	5 (17.9%)
	iii) Team	33 (23.6%)	5 (9.4%)	5 (17.9%)
	iv) Individual	27 (19.3%)	26 (49.0%)	9 (32.1%)
Stage of ‘Burnout’	i) Preventing	111 (79.3%)	40 (75.5%)	21 (75%)
	ii) Addressing	18 (12.9%)	8 (15.1%)	3 (10.7%)
	iii) Supporting Recovery	11 (7.8%)	5 (9.4%)	4 (14.3%)
Topic	i) Promoting Mental Health	80 (57.1%)	35 (66.0%)	19 (67.9%)
	ii) Managing Workload	12 (8.6%)	4 (7.5%)	2 (7.1%)
	iii) Handling Conflict, Bullying & Harassment	19 (13.6%)	5 (9.4%)	3 (10.7%)
	iv)Addressing/Confronting Discrimination	16 (11.4%)	4 (7.5%)	2 (7.1%)
	v) Reducing Stigma & Facilitating Disclosure	10 (7.1%)	5 (9.4%)	2 (7.1%)
Audience	i) Pharmacy	*Not Recorded for Original R&I*	0	0
	ii) Healthcare		37 (69.8%)	17 (60.7%)
	iii) Occupations at risk		5 (9.4%)	5 (17.9%)
	iv) Societal		11 (20.8%)	6 (21.4%)
Medium	i) Electronic		37 (69.8%)	19 (67.9%)
	ii) Face to Face		7 (13.2%)	4 (14.3%)
	iii) Telephone		1 (1.9%)	1 (3.6%)
	iv) Virtual		7 (13.2%)	3 (10.7%)
	v) Unknown⁎		1 (1.9%)	1 (3.6%)
Engagement Type⁎	i) Interactive		14 (26.4%)	8 (28.6%)
	ii) Passive		38 (71.7%)	19 (67.9%)
Format⁎	i) Facilitated Group		8 (15.1%)	5 (17.9%)
	ii) One on one		4 (7.5%)	2 (7.1%)
	iii) Chatbot		1 (1.9%)	0
	iv) Multiple offerings		8 (15.1%)	4 (14.3%)
	v) Print		26 (49.0%)	13 (46.4%)
	vi) Audio		1 (1.9%)	0
	vii) Video		4 (7.5%)	3 (10.7%)
Anonymized Intervention/Resource⁎	i) Anonymous		37 (69.8%)	18 (64.3%)
	ii) Not Anonymous		15 (28.3%)	9 (32.1%)

⁎ One unique R&I behind pay-wall and so unable to verify details on four variables (not added up to 100%).

Overall, of the highly-graded 53 R&I, 40 (75.5%) were focused on preventing burnout, 8 (15.1%) on addressing burnout and 5 (9.4%) on supporting recovering from burnout. The group's final review of these 53 R&I retained 28 for use within an orchestrated national plan. At every stage of the screening and review process, the majority of R&I were focused on ‘preventing burnout’ and ‘promoting mental health’ at ≥70% and ≥ 50%, respectively. In the latter two rounds the majority of R&I were characterised as targeting a *healthcare audience* of prospective users, through an *electronic* medium, with *anonymity* being presumably secured, albeit in a *passive* format of engagement (>60% for all).

## 4 Discussion

Knowledge translation of the CHWN Toolkit was an expedited process to produce a selection of R&I potentially effective for the profession of pharmacy, particularly community pharmacy. It did not include critical appraisal of the research but rather sought to use knowledge mobilised through CHWN's research. The work did not attempt to correlate R&I characteristics or variables to identify relationships (level, stage of burnout and topic etc.). This work is therefore descriptive and exploratory and not statistically analytic. Users may find value in performing their own sub-analyses of data for the purposes of describing gaps in R&I and informing new R&I design and content. (See **Supplemental Appendices**). The Toolkit has been updated by CHWN to include more R&I since this project concluded.

The CHWN Toolkit reduced the working group's need to perform a systematic literature review and emerged at an opportune point when resources were being sought for a profession suffering declines in psychological health and wellbeing. Other health professions may wish to build on the approach described here in their knowledge translation efforts to address burnout/occupational psychological health and wellbeing within their own ranks. International organisations with a mandate to improve or maintain occupational psychological health and wellbeing may also find this work useful in conjunction with expanding their literature searching for R&I in languages other than English.^44^

Within the 28 selected R&I, some were multi-faceted, functioning as a suite of resources or advice on one or more topics (i.e. the PRN - “Pause, Resent and Nourish” toolkit or the “Violence, Aggression and Responsive Behaviours” toolkit).^45^^,^^46^ Some R&I were higher production short video series on mental health in the workplace that provided techniques ranging from mindfulness techniques to mental health in the workplace.^47^^,^^48^ Some of the more complex R&I were either web-interactive (such as an anonymous online forum) or offered sustained programming that can be customised for individuals within or outside workplaces for the profession (such as peer-support programs or employee assistance programs). These R&I require significant adaptation and resourcing on the part of multiple stakeholders prior to implementation within the profession.^49^

### 4.1 Systems and individual R&I were predominant

Organisational and team level R&I were less represented overall in the final set of R&I. This divergence is related to the community pharmacy setting being unique from most healthcare organisations and teams. The organisation and team level R&I located within institutional or broad healthcare employment settings would be unlikely to receive high grades given the significant tailoring necessary to adapt it for community pharmacy. Individual level interventions are applicable and effective for many types of professionals (not just pharmacy) and therefore more likely to receive a high grade. Individual R&I however, do not address the underlying, structural bases that contribute to declining health and wellbeing. Any benefit from this level of R&I may be short-lived, small-scale and risk diverting attention from the profound and durable impact systems and organisational R&I can convey.

Systems R&I are prominent in the selected 28 R&I. They are a cache for pharmacy leadership (advocacy agencies, regulators, and pharmacy corporations) to research, refine, implement, and monitor the impact of R&I on the pharmacy workforce. The high number of managers working within discrete flat organisational structures of each pharmacy mean significant leadership creativity and perseverance to ensure systems-level R&I permeate these organisational units and provide sufficient resources (time, expertise, and funding). Differentiation between organisational and team R&I may not be possible or desirable given the flat organisational structures inherent in community pharmacy.

### 4.2 ‘Prevention of burnout’ R&I were predominant

This statistic (75%) is consistent with the rate encountered within the larger CHWN Toolkit. It may be reflective of the knowledge gaps in professional populations experiencing or recovering from the phenomena or it may be associated with the high degree of complexity and resourcing needed to address or recover from burnout. Although an ounce of prevention is worth a pound of cure, there is an ongoing need to ensure the returning workforce is also supported consistently across the country with evidence-based R&I.

### 4.3 ‘Promotion of mental health’ R&I were predominant

These R&I (67.9%) considerably outnumbered R&I targeted at ‘Managing Workload’, ‘Handling Conflict, Bullying and Harassment’, ‘Addressing/Confronting Discrimination’ or ‘Reducing Stigma and Facilitating Disclosure’. This represents an opportunity for designing and building out R&I focussing on these distinct factors associated with occupational psychological health and wellbeing. Pharmacy professionals are particularly subjected to these factors.^28^ The public visibility/accessibility of pharmacy professionals present considerable risk of conflict/harassment during daily practice. High visibility coupled with the rich diversity within the profession predispose minority groups to the potential stress and trauma of discrimination.

### 4.4 Lack of pharmacy specific R&I

The absence of research describing R&I in pharmacy indicates a need for knowledge mobilisation in this aspect of Social, Behavioural and Administrative Pharmacy and/or Occupational Health. Research is recommended to: 1) Identify the factors that facilitate or stymie psychological health and wellbeing within pharmacy practice (discovery research) and 2) Measure the effectiveness of R&I targeting these identified factors (program evaluation).

The 28 R&I identified require significant adaptation and research prior to piloting or as a means of monitoring outcomes to ensure success. These R&I may be a starting point for researchers with interest in occupational psychological health and wellbeing. While nursing and medicine will find significant research within their professions to potentially justify direct implementation of many CHWN Toolkit R&I, other professions will, like pharmacy, need to base their workforce support on exploratory research within their respective fields. Whether community pharmacy decision makers and professionals will hesitate to implement resource-intense R&I that have only shown success or feasibility in non-pharmacy health professions is unknown.

### 4.5 Electronic delivery of R&I was predominant

More than half (67.9%) of R&I examined employed this medium, which mirrors that of the CHWN Toolkit. Electronic R&I are accessible for most pharmacy professionals and leaders given their familiarity with information technology. It is important to accommodate different preferences and needs of professionals and include person-to-person R&I. Further investigation is merited to understand whether the number of face-to-face (4), telephone (1) and virtual (3) R&I sufficiently meets the needs of the profession.

### 4.6 Passive R&I was predominant

The proportion of passive R&I (67.9%) matched the electronic delivery rate (67.9%) with much overlap between the two categories (i.e. many R&I were *both* electronic and passive) suggesting that the electronic medium may be more conducive to delivering passive engagement types of R&I. While consistent with the overall rate within the CHWN Toolkit, the dominance of passive R&I may fill general knowledge gaps relating to burnout and occupational psychological health and wellness. However, passive learning is not congruent with adult learning principles and theory. R&I that include interactive engagement with material and/or peers increases the potential for meaningful learning and may present greater opportunity for skill and attitude development that underlies change.

### 4.7 Anonymity is often maintained in R&I

By virtue of the medium, engagement type or format, the high rate (64.3%) of anonymous R&I responds to the stigma that pharmacy professionals face in acknowledging burnout or poor psychological health and associated worry about professional relationships and employment levels. Engagement in active, in-person R&I often requires the individual trade their anonymity for a personalised intervention involving human relationship(s). Further investigation is needed to understand if the 9 (32.2%) non-anonymous R&I present barriers to engagement.

### 4.8 Study limitations

This study has some important limitations. Firstly, the process of grading R&I was not formally validated for inter-rater reliability. This could result in some degree of variance in the results if the study were replicated by others. Secondly, an exhaustive search to capture R&I beyond the originals compiled by the CHWN was not performed. Thirdly, this work was not updated after CHWN expanded on their original R&I list. As such, the authors are aware of at least one R&I that has since been added that is exclusive to female empowerment and leadership in pharmacy.^50^ Lastly, as previously mentioned, the authors did not conduct a systematic review of evidence on the ‘types’ of R&I.

## 5 Conclusion

The majority of the final 28 R&I graded as relevant and potentially useful to support the pharmacy profession targeted systems and individual levels, aimed at preventing burnout and promoting mental health. These R&I were often delivered electronically and passively exposed the participant to content. None of the 28 selected R&I were based on evidence generated from within the pharmacy profession. Nonetheless, these R&I, with appropriate adaptation, may serve as an initial basis for an orchestrated national plan to maintain pharmacy's professional workforce and quality of patient care through maintaining or improving occupational psychological health and wellbeing.

## Artificial intelligence disclosure

The authors did not use or collaborate with any Artificial Intelligence application, software, program, or conscious entity in the production of this work. The authors relied solely on the frailty of the human mind in each of the research tasks.

## Declaration of Competing Interest

The authors declare no financial or personal conflicts of interest as it relates to this work.

## Appendices Supplementary data

Canadian Health Workforce Network (CHWN) 'burnout' Toolkit Image 1

Data charting results workbook Image 2

## Footnotes

2 The Toolkit has been redeveloped subsequent to this work into a publicly available webpage: https://www.mhcaretoolkit.ca/burnout-toolkit%p
