# Supplementary material for: Resources and interventions to support psychological health and wellbeing in the pharmacy workforce: Analysis and use of a health worker ‘burnout’ toolkit
Source: Explor Res Clin Soc Pharm. 2023 Nov 3;12:100359. doi: 10.1016/j.rcsop.2023.100359 (PMC10641757; doi:10.1016/j.rcsop.2023.100359)
Supplement: Supplementary file 1 — Canadian Health Workforce Network (CHWN) 'burnout' Toolkit [file mmc1.docx]

**CIHR – Healthcare Worker Burnout Toolkit – Draft Nov. 15^th^**

# **Definitions**

## **Target of Intervention**

- **System level** tools, toolkits and interventions target policies at an association/union level, collective agreements, government action or advocacy.
- **Organization level** tools, toolkits and interventions focus on policies within an organization, leadership development and training opportunities within the work environment.
- **Team/work unit level** tools, toolkits and interventions focus on peer support, mentorship, and leadership within a group setting.
- **Individual level** tools, toolkits and interventions focus on worker empowerment, changes in work culture or aimed at leadership within a division.

**Types/Levels of Burnout**

- **Burnout** – “Burn-out is a syndrome conceptualized as resulting from chronic workplace stress that has not been successfully managed. It is characterized by three dimensions: *feelings of energy depletion or exhaustion*; *increased mental distance from one’s job*, or *feelings of negativism or cynicism related to one's job; and reduced professional efficacy*.” ^^[[1]](#endnote-1)^^
- **Preventing Burnout** – Tools, toolkits and interventions aimed “at reducing known risk factors among all employees, in order to prevent burnout from developing”.^^[[2]](#endnote-2)^^ *Primary - prevention and promotion of mental health and psychological well-being in the workplace (e.g., job redesign, workplace health promotion).*
- **Addressing Burnout** – Tools, toolkits and interventions aimed at healthcare workers evaluated to be at a high risk (i.e., those who have experienced or are currently experiencing excessive and prolonged work-related stress).^^[[3]](#endnote-3)^^ *Secondary–acute treatment - active training and educational programming/ interventions/ (e.g., coping skills training; stress management programs)*
- **Supporting burnout recovery** – tools, toolkits and interventions aimed at workplace accommodations for healthcare workers who were off work after experiencing excessive and prolonged stress and burnout to facilitate their recovery/treatment and return to work.^^[[4]](#endnote-4)^,^[[5]](#endnote-5)^^ *Tertiary – recovery and rehabilitation (e.g., EAP, RTW program).*

## **Cross-Cutting Workplace Influences on Burnout**

- **Workload management** – A state of well-being that a healthcare worker can reach or can set as a goal to allow them to manage effectively multiple responsibilities at work.^[[6]](#endnote-6)^
- **Conflict, bullying & harassment** – Any inappropriate action by a person towards a worker “that he or she knew or reasonably ought to have known would cause that worker to be humiliated or intimidated”. ^[[7]](#endnote-7)^
- **Discrimination** – “Occurs when an employee or group of employees is treated differently, negatively or adversely because of their gender, race, color, culture, nationality, ethnic origin, religion, age, disability, marital status, pregnancy, sexual orientation, trade union membership or any other prohibited grounds of discrimination.”^[[8]](#endnote-8)^
- **Stigma** – A process that involves awareness and/or endorsement of stereotypes, labelling, separation, prejudice and discrimination in a context in which social, economic or political power is exercised to the detriment of members of a social group.^[[9]](#endnote-9)^
- **Disclosure** – “Telling a supervisor or member of your workplace’s human resources team about a diagnosis of any health problem (including MH issues).”^[[10]](#endnote-10)^

#
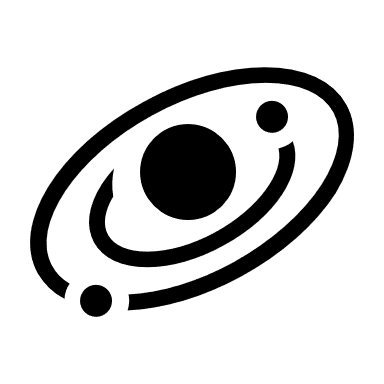
System Level Tools, Toolkits & Interventions

* **System** level toolkits and interventions target policies at an association/union level, collective agreements, government action or advocacy

| *For Gender/ Intersectional Informed | **Promoting Mental Health** | **Managing Workload** | **Handling Conflict, Bullying & Harassment** | **Confronting Discrimination** | **Reducing Stigma & Facilitating Disclosure** |
| --- | --- | --- | --- | --- | --- |
| **Preventing Burnout** | [**Addressing Mental Health in the Workplace**](https://www.youtube.com/playlist?list=PL2NuAPXp8ohZmoVaECl6sRiV9lQ25XkId)  – Learn about the [National Standard of Canada for Psychological Health and Safety in the Workplace (the Standard)](https://mentalhealthcommission.ca/national-standard/) – 14 videos relating to psychological health and safety (Mental Health Commission of Canada) [Canada]. | [**Disconnecting from work policy**](https://www.ontario.ca/document/your-guide-employment-standards-act-0/written-policy-disconnecting-from-work)  – Employers that employ 25 or more employees in Ontario on January 1 of any year are required to have a written policy on disconnecting from work (i.e., not engaging in work-related communications, including emails, telephone calls, video calls or sending or reviewing other messages, to be free from the performance of work)  [Canada, Ontario]. | [**Workplace harassment and violence risk assessment tool**](https://www.canada.ca/en/employment-social-development/programs/workplace-health-safety/harassment-violence-prevention/risk-assessment-tool.html) – A sample workplace harassment and risk assessment form created by the Government of Canada [Canada]. | **[Ontario Health’s Equity,](https://www.ontariohealth.ca/sites/ontariohealth/files/2020-12/Equity%20Framework.pdf)**  **[Inclusion, Diversity and](https://www.ontariohealth.ca/sites/ontariohealth/files/2020-12/Equity%20Framework.pdf)**  **[Anti-Racism Framework](https://www.ontariohealth.ca/sites/ontariohealth/files/2020-12/Equity%20Framework.pdf)**  **–** A tool to build an organizational culture focused on equity, inclusion, diversity and anti-racism, and to contribute to better outcomes for patients, families and providers within the health system (Ontario Health) [Canada, Ontario]. | [**Opening Minds**](https://mentalhealthcommission.ca/opening-minds/)  – The largest systematic effort in Canadian history focused on ***reducing stigma*** related to mental illness (Mental Health Commission of Canada) [Canada]. |
|  | [**Creating a Safe Space**](https://www.patientsafetyinstitute.ca/en/toolsResources/Creating-a-Safe-Space-Psychological-Safety-of-Healthcare-Workers/Documents/Manuscript%20Documents/4_Canadian%20Best%20Practices%20for%20peer%20to%20peer%20support%20programs.pdf)  – This manual provides a comprehensive overview of what peer support is available in Canada and internationally. Most importantly, it provides best practice guidelines, tools and resources, to assist policy makers, accreditation bodies, regulators and healthcare leaders assess what healthcare workers need in terms of support, and to create PSPs to help them improve their emotional well-being and allow them to provide the best and safest care to their patients [Canada]. |  | [**Violence, Aggression & Responsive Behaviours Toolkit**](https://workplace-violence.ca/tools/)  – Validated, consensus-based resources for workplace violence prevention in hospitals, long-term care, home and community care and emergency medical services (Public Services Health & Safety Association) [Canada, Ontario]. | [**Principles and strategies for anti-racism interventions in healthcare settings**](https://www.ncbi.nlm.nih.gov/pmc/articles/PMC8000324/)  – A conceptual model (*see Figure 2*) that depicts the process, principles, and strategies for consideration when implementing anti-racism interventions in healthcare settings (scholarly publication) [Canada, Ontario]. | [**Like Minds, Like Mine**](https://www.likeminds.org.nz/about/) – A public awareness programme to increase social inclusion and end discrimination towards people with experience of mental illness or distress through public awareness campaigns, community projects and research.  [New Zealand].  [**See me**](https://www.seemescotland.org/about-see-me/)  – A public awareness programme to tackle mental health stigma and discrimination  [Scotland] |
|  | [**Mental Health in the Workplace**](https://ccdi.ca/media/3612/educational-resources-mental-health-en.pdf)  – A guide that provides educational resources to learn how to encourage and drive conversations around mental health in the workplace (Canadian Centre for Diversity and Inclusion) (***intersectionally focused***) [Canada]. |  | [**Workplace violence prevention in health care: A guide to the law for hospitals, long-term care homes and home care**](https://www.ontario.ca/page/workplace-violence-prevention-health-care-guide-law-hospitals-long-term-care-homes-and-home-care#:~:text=Health%20care%20workers%20have%20the%20right%20to%20do,violence%20%E2%80%94%20one%20incident%20is%20one%20too%20many.)  – Aims to help employers, supervisors and workers understand their obligations under the *Occupational Health and Safety Act (OHSA)* and *O. Reg. 67/93* *– Health Care and Residential Facilities Regulation*, including workplace violence prevention (Government of Ontario)  [Canada, Ontario]. | [**Dismantling Anti-Black Racism: A Toolkit for Health Professionals**](https://healthsci.queensu.ca/sites/opdes/files/modules/dismantling-anti-black-racism/#/)  – A module designed to further the understanding of anti-Black racism in the healthcare professions and to provide resources to combat and address racism in learning and teaching spaces (Queen’s University)  [Canada, Ontario]. |  |
|  | [**Evidence-based practices support the Heart of Safety: Declaration of Principles**](https://www.vocera.com/blog/evidence-based-practices-support-ceo-coalitions-declaration-principles)  – In 2021, the CEO Coalition engaged the Institute for Healthcare Improvement (IHI) to research evidence- and experience-based practices that support the principles outlined in its Heart of Safety: Declaration of Principles. This blog post outlines those practices *(which include psychological health and safety)* [United States] (See also: [Bridget Duffy, 2021](https://journals.sagepub.com/doi/pdf/10.1177/08404704211048806)). |  | [**Prevention and management of violence and aggression in health services**](https://content-v2.api.worksafe.vic.gov.au/sites/default/files/2018-06/ISBN-Prevention-and-management-of-violence-and-aggression-health-services-2017-06.pdf) – A guidebook for employers on how to prevent and manage occupational violence and aggression towards health workers (WorkSafe Victoria) [Australia]. | [**The Nokiiwin Workplace Wellness Toolkit**](https://www.nokiiwin.com/upload/documents/health-and-safety/c65-employee-toolkit.pdf)  – A response to the growing need for education, training, and skills for First Nations employers to create healthier and safer workplaces (Nokiiwin Tribal Council) [Canada]. |  |
|  | [**National Plan for health workforce well-being**](https://nam.edu/initiatives/clinician-resilience-and-well-being/national-plan-for-health-workforce-well-being/)  – A peer reviewed publication that intends to drive collective action to strengthen health workforce wellbeing and restore the health of the nation. It calls on multiple actors – including health care and public health leaders, government, payers, industry, educators, and leaders in other sectors – to help drive policy and systems change (National Academy of Medicine)  [United States]. |  | [**Position Statement on Bullying**](https://www.ontariomidwives.ca/aom-position-statement-bullying)  – A clear stance on harassment, bullying and any behaviour that undermines midwives in any work setting. An invitation to denounce bullying in practice groups, birth centres, hospitals and all other places of work and life and address it through a four-prong approach (Association of Ontario  Midwives) [Canada, Ontario]. | [**Diversity, Equity & Inclusion Position Statement**](https://www.ontariomidwives.ca/diversity-equity-inclusion-position-statement)  – A clear stance on diversity, equity and inclusion (Association of Ontario Midwives)  [Canada, Ontario].  [**Racial Justice Position Statement**](https://www.ontariomidwives.ca/racial-justice-position-statement)  – A clear stance on racial justice (Association of Ontario Midwives)  [Canada, Ontario]. |  |
|  | [**Magnet Recognition Program**](https://www.nursingworld.org/organizational-programs/magnet/about-magnet/) – A designation from the American Nurses Credentialing Center that recognizes nursing excellence. Hospitals achieve Magnet Recognition status for quality patient care and innovations in professional nursing practice. It is considered the highest recognition for a hospital nursing department. An [analysis of Magnet hospitals](https://www.mdpi.com/1660-4601/18/2/610/htm) found that they were associated with a reduction in nurse burnout [United States] (See also: [Canada’s Magnet Hospital](https://www.sinaihealth.ca/home/canadas-magnet-hospital/#:~:text=The%20Magnet%20Recognition%20Program%20provides%20a%20roadmap%20to,which%20leads%20to%20greater%20autonomy%20at%20the%20bedside.)). |  | [**Issue Brief: Addressing workplace violence in leading health systems**](https://academynet.com/knowledge-article/issue-brief-addressing-and-preventing-workplace-violence-leading-health-systems) –  In 2022, The Academy conducted research on workplace violence (WPV) in healthcare, including secondary data collection, applied literature review, and qualitative interviews with frontline healthcare team members. This brief outlines the impact of WPV on healthcare professionals and the entire healthcare system, and offers a maturity model that leaders can use to advance efforts to address the WPV epidemic (Health Management Academy)  [United States]. |  |  |
|  | [**CEO Coalition 2022 Research Report: Restoring a Safe and Thriving Healthcare Workforce**](https://www.ceocoalition.com/wp-content/uploads/2022/09/CEO-Coalition_2022-Research-Report_Retaining-a-Safe-and-Vibrant-Healthcare-Workforce.pdf) – This report inventories the practices and solutions leading health systems are implementing to support a safe and thriving workforce. It addresses both safety and recruitment/ retention. The report includes highlighted stories of leading practices that are making a difference for team member safety and well-being [United states]. |  |  |  |  |
|  | [**Physician Wellness Hub**](https://www.cma.ca/physician-wellness-hub) – This webpage/toolkit provides reliable wellness tools and resources to empower physicians, medical learners, leaders and educators to create change in the culture of medicine (***multileveled***) (Canadian Medical Association) [Canada]. |  |  |  |  |
| **Addressing Burnout** | **Provincial wellness/mental health support programs for physicians:**  [**Physician Health Program (PHP)**](https://www.mspei.org/salary-and-employee-benefits/salary-and-employee-benefits-physician-health-program/) – Medical Society of Prince Edward Island  [**Physician Health Program**](https://php.oma.org/about-php/) – Ontario Medical Association  [**Physician and Family Support Program**](https://doctorsmanitoba.ca/physician-health/wellness-programs/physician-family-support-program) – Doctors Manitoba  [**Physician Health Program**](https://www.sma.sk.ca/programs/44/physician-health-program.html) –Saskatchewan Medical Association  [**Professional Support Program**](https://doctorsns.com/benefits/professional-support) **–**Doctors Nova Scotia |  | [**Enough is Enough. Putting a Stop to Violence in the Health Care Sector**](https://nursesunions.ca/wp-content/uploads/2017/05/CFNU_Enough-is-Enough_June1_FINALlow.pdf)  – A discussion paper that aims to catalyze a national discussion on violence in health care; brings together the disparate stories from coast to coast, highlighting its broad and pervasive impacts (Canadian Federation of Nurses Union)  [Canada]. |  |  |
|  | **Wellness/mental health support programs for dentists:**  [**Members Assistance Program (MAP)**](https://www.cdspi.com/members-assistance-program/) – Canadian Dental Association and participating Provincial and Territorial Dental Associations  [**Dentist Wellness Program**](https://www.bcdental.org/membership-benefits/dental-office-wellness/) – The Voice of BC Dentists, British Columbia Dental Association  [**Benevolence Program**](https://www.oda.ca/member-resources/wellness-support-services/benevolence-program/) – Ontario Dental Association |  |  |  |  |
| **Supporting Burnout Recovery** | [**Policy and Procedures on the Accommodation of Mental Illness**](https://www.chrc-ccdp.gc.ca/sites/default/files/policy_mental_illness_en_1.pdf)  – Outlines the accommodation process and provides guidance to help managers and supervisors take proactive steps to ensure employees with a mental illness are offered appropriate accommodation when necessary, with aim of creating a work environment that supports health and well-being of all employees and that contributes to preventing mental illness (Canadian Human Rights Commission)  [Canada]. |  |  |  |  |
|  | [**Locum Protocols**](https://nsdental.org/wp-content/uploads/2019/05/Enhancing-the-Wellness-of-Canadian-Dentists.pdf)  – Ensure that dentists who must withdraw from practice to undergo treatment for mental health or addiction reasons keep their practice viable in their absence  [Canada, British Columbia].  [**Future Insurability**](https://nsdental.org/wp-content/uploads/2019/05/Enhancing-the-Wellness-of-Canadian-Dentists.pdf)  – Need for policy to address and protect the future insurability of the dentist for short- or long-term disability once they have undergone rehabilitation for an addiction or a mental health problem (p. 7)  [Canada, British Columbia]. |  |  |  |  |
|  | [**Rehabilitation and Wellness Services**](https://www.oda.ca/member-resources/wellness-support-services/rehabilitation-and-wellness-services/) – Dentists living with addictions can take advantage of a range of rehabilitative services for health-care providers (Ontario Dental Association) [Canada, Ontario]. |  |  |  |  |

#
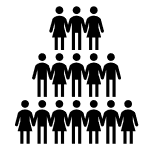
Organization Level Tools, Toolkits & Interventions

* **Organization** level toolkits and interventions focus on policies within an organization, leadership development and training opportunities within the work environment.

| *****For Gender/ Intersectional Informed | **Promoting Mental Health** | **Managing Workload** | **Handling Conflict, Bullying & Harassment** | **Confronting Discrimination** | **Reducing Stigma & Facilitating Disclosure** |
| --- | --- | --- | --- | --- | --- |
| **Preventing Burnout** | [**Beating burnout in the NHS**](https://www.nhsemployers.org/articles/beating-burnout-nhs)  – This guidance intends to support leaders in the NHS, including health and wellbeing leads and managers, who all play an important role in beating staff burnout (National Health Service)  [United Kingdom] | [*** Time bank/time-banking system**](https://publications.aap.org/pediatrics/article/148/Supplement%202/e2021051440H/183786/Innovative-Wellness-Models-to-Support-Advancement?autologincheck=redirected?nfToken=00000000-0000-0000-0000-000000000000)  – A strategy that offers credit to physicians for uncompensated work (such as mentoring or serving on committees); these credits could then be used for services or assistance that facilitate better work-life harmony  [United States] | [**Workplace Violence and Aggression Prevention Program**](https://continuingcaresafety.ca/workplace-violence-aggression/) – Provides information, framework, training and resources needed to incorporate an effective workplace violence prevention program into the health and safety management system  [Canada] | [**A Toolkit to Advance Racial Health Equity in Primary Care Improvement**](https://www.chcf.org/publication/toolkit-racial-equity-primary-care-improvement/)  – Offers seven concrete opportunities to act and advance health equity  [United States] | [**Think Mental Health**](http://thinkmentalhealth.ca/watch-videos/)  – A series of videos on mental health, courtesy of Ottawa Public Health  [Canada]. |
|  | [**The rising cost of living: key facts, employer response and resources**](https://www.nhsemployers.org/sites/default/files/2022-09/The-rising-cost-of-living-information-pack.pdf)  – An information pack that provides a summary of the areas that employers may wish to explore to support health care staff with the rising cost of living (***intersectionally focused***). It recognizes that workplace and external pressures such as financial struggles can both contribute to mental health issues, including burnout. (See also: [The rising cost of living \| NHS Employers](https://www.nhsemployers.org/publications/rising-cost-living))  [United Kingdom]. | [**Getting Rid of Stupid Stuff Program**](https://edhub.ama-assn.org/steps-forward/module/2757858) – Clinical staff who use the electronic health record system identify documentation and other practice requirements that should be eliminated, changed or modified. (See also: [Getting Rid of Stupid Stuff - Building Trust](https://buildingtrust.org/practice/getting-rid-of-stupid-stuff/)).  [Hawai’I United States] | [**Violence Prevention Strategies**](https://ochu.on.ca/wp-content/uploads/2019/08/2017-11-27-Assaulted-and-Unheard-Violence-Against-Healthcare-Staff-New-Solutions-Article.pdf) – ***See Table 1*** (pp. 17-18) for primary, secondary and tertiary prevention strategies that were recommended by health workers from diverse communities (research participants) (scholarly publication)  [Canada, Ontario]. | [**Centering a more holistic view of workforce safety**](https://www.ihi.org/communities/blogs/centering-a-more-holistic-view-of-workforce-safety) – In this Institute for Health Improvement blog post, the Institute’s research team outlines how to improve safety and well-being by gathering and examining data on team member safety incidents, listening to frontline staff about safety needs and solutions, making a culture of safety a priority, and stratifying workforce safety data to build a baseline for equity [United States]. | [**Key ingredients of anti-stigma programmes for healthcare providers**](https://link.springer.com/content/pdf/10.1007/s00127-017-1341-9.pdf)  ***–***Face to face stigma reduction training for healthcare providers was conducted within Canada’s Opening Minds anti-stigma programme. Thirty-seven contact-based education programmes were evaluated to identify the key ingredients associated with attitude change. ***Table 1*** (p. 252) provides a summary of these findings (scholarly publication). |
|  | [**StressAssess**](https://stressassess.ca/sa_doc/about.php?lang=en)  – An online survey tool designed for workers and managers who are interested in assessing psychosocial hazards in the workplace (Occupational Health Clinics for Ontario Workers)  [Canada, Ontario]. | [**Shared service schedule**](https://pubmed.ncbi.nlm.nih.gov/28362643/)  – An alternative ICU staffing model predicted to improve continuity of care while increasing free weekends and continuity of uninterrupted nonclinical weeks for attendings (instead of a standard “7 days on schedule”) (scholarly publication). | [**The University of British Columbia Faculty of Dentistry Sexual Violence Awareness Page**](https://www.dentistry.ubc.ca/education/students/student-support/sexual-violence-awareness/)  – Provides information on sexual violence and different resources and supports available to students from the Faculty of Dentistry  [Canada, British Columbia]. | [**Equity, diversity and inclusion learning path for executives**](https://www.csps-efpc.gc.ca/diversity-inclusion-eng.aspx)  **–** Resources to assist executives in developing the awareness and competencies required for a diverse and inclusive workplace (Government of Canada)  [Canada]. |  |
|  | [**Health and wellbeing champions**](https://www.nhsemployers.org/articles/health-and-wellbeing-champions)  – People at all levels of the NHS who promote, identify, and signpost ways to support the wellbeing of their colleagues [United Kingdom].  [**Scholars of Wellness: A faculty development program to create wellness champions**](https://www.youtube.com/watch?v=1Rar6kT8f6o)  **–** A video that presents an award-winning faculty development initiative which can be adopted by organizations to help create a critical mass of faculty wellness champions that can advance well-being at the organizational level (American Medical Association)  [United States].  [**Wellbeing Guardians**](https://learninghub.leadershipacademy.nhs.uk/wp-content/uploads/dlm_uploads/2021/09/B0189-Wellbeing-Guardian-Implementation-Guidance-Final-for-publishing.pdf)  **–** A guidance for introducing the Wellbeing Guardian role in healthcare organizations; the Wellbeing Guardian will seek to assure and continue to reassure the board that their organization is a wellbeing organization and a healthy workplace in which NHS staff and learners can work and thrive  [United Kingdom]. | [**Workload management: A guide for managers**](https://www.forgov.qld.gov.au/__data/assets/pdf_file/0025/182491/workload-management-guide-for-managers.pdf)  – Developed under the State Government Departments’ Certified Agreement to assist managers’ work with employees to design work practices that promote wellbeing, work-life balance and prevent harm  [Australia]. | **[Individual Client Risk](https://workplace-violence.ca/wp-content/uploads/2017/06/VPRASEEN0417-ICRA-Toolkit-Resource-Manual-V1.3-2017.12.12-1.pdf)**  **[Assessment Toolkit for Health Care Settings](https://workplace-violence.ca/wp-content/uploads/2017/06/VPRASEEN0417-ICRA-Toolkit-Resource-Manual-V1.3-2017.12.12-1.pdf)**  – Helps to identify behaviours and triggers associated with increased risk of violence so prevention measures for staff and the client may be taken  [Canada, Ontario]. | [**Improving Workplace Culture through Evidence-Based Diversity, Equity and Inclusion Practices**](https://www.wharton.upenn.edu/wp-content/uploads/2021/05/Applied-Insights-Lab-Report.pdf) **–** Research report with key findings and recommendations of evidence-based diversity, equity and inclusion practices (Wharton School, University of Pennsylvania)  [United States]. |  |
|  | [**Guarding Minds at Work**](https://www.guardingmindsatwork.ca/)  – A comprehensive formal framework for helping employers assess and address psychological health and safety in the workplace  [Canada]. | [**Handbook of Occupational Hazards and Controls for Dental Worker**s](https://open.alberta.ca/dataset/110d20f0-3599-4605-9269-10ee9d8f0f05/resource/ebee312e-cc50-42dc-b5c0-3cd341759c62/download/ohs-wsa-handbook-dental-workers.pdf)  – Provides information for employers about control strategies for commonly occurring psychological hazards, including ways to reduce the impact of increased workloads (e.g., allow workers to say “no” to overtime without repercussions (p. 48) [Canada, Alberta]. |  |  |  |
|  | [**Leaders supporting leaders**](https://www.ncbi.nlm.nih.gov/pmc/articles/PMC9127620/)  – Eight actions to build resilience and psychologically healthy workplaces during the pandemic and beyond  [Canada]. | [**Bedside Learning Coordinator (BLC)**](https://qualitysafety.bmj.com/content/qhc/30/6/509.full.pdf)  – A role created to source ideas for improvement from front-line staff and take them to decision-makers who can act on them to improve standard work, and to communicate changes back to front-line staff and monitor success of implementation  [United Kingdom]. | [**Managing disruptive behaviour in the healthcare workforce**](https://hqca.ca/wp-content/uploads/2021/10/Disruptive_Behaviour_Toolkit_042413.pdf)  – This Resource Toolkit provides templates, checklists, tools and other sample documents that organizations or workplaces can use to develop materials to support a behaviour-related initiative  [Canada, Alberta]. |  |  |
|  | [**Innovative Wellness Models to Support Advancement and Retention Among Women Physicians**](https://publications.aap.org/pediatrics/article/148/Supplement%202/e2021051440H/183786/Innovative-Wellness-Models-to-Support-Advancement?autologincheck=redirected?nfToken=00000000-0000-0000-0000-000000000000)  – This scholarly publication presents three wellness-oriented models to promote the professional fulfillment and well-being of diverse groups of women physicians (***intersectionally focused***)  [United States]. |  | **[Workplace Violence in Hospitals:](https://oahhs.org/assets/documents/documents/safety/WPV/Toolkit%20all%20Sections%20with%20PDF%20index%20(no%20tools).pdf)**  **[A Toolkit for Prevention & Management](https://oahhs.org/assets/documents/documents/safety/WPV/Toolkit%20all%20Sections%20with%20PDF%20index%20(no%20tools).pdf)**  **–** Provides practical tools, resources and information that can be used by hospitals, and adapted for a range of other health care settings, to develop and sustain effective violence prevention programs  [United States]. |  |  |
|  | [**Resilience Toolkit for Nurse Leaders: Interventions to Build a Resilient Culture**](https://www.sciencedirect.com/science/article/abs/pii/S1541461218302830)  – ***Figure 2*** (p. 47) presents a multileveled nurse leader toolkit which contains a variety of resilience interventions to develop a culture of health and well-being and support professional joy  [United States]. |  | [**Manager’s Toolkit – Leading in a hybrid work environment**](https://mentalhealthcommission.ca/wp-content/uploads/2022/04/Managers-Toolkit-%E2%80%93-Leading-in-a-Hybrid-Work-Environment-1.pdf)  – A resource for managers looking to minimize the virtual divide in the workplace. Provides actionable guidelines for managing workplace conflicts and maintaining social cohesion (Mental Health Commission of Canada) [Canada]. |  |  |
|  | [**Occupational hazards in the health sector**](https://www.who.int/tools/occupational-hazards-in-health-sector/)  – This e-tool is intended for use by people in charge of occupational health and safety for health workers at the national, subnational and facility levels and for health workers who want to know what World Health Organization and the International Labour Organization recommend for the protection of their health and safety (***multileveled***)  [International]. |  |  |  |  |
|  | [**Michigan Medicine Burnout Toolkit**](https://wellnessoffice.med.umich.edu/knowledge-hub/tools-resources/burnout-toolkit)  – Facilitates learning about burnout, its contributors and key strategies to address it for leaders, faculty, staff, and learners (***multileveled***) (Wellness Office, University of Michigan)  [United States]. |  |  |  |  |
|  | [**Addressing Burnout – A Training Program for Healthcare Leaders**](https://medsites.vumc.org/centerforprofessionalhealthbrvanderbiltuniversitymedicalcenter/addressing-burnout-training-program)  **–** Trains leaders within departments and divisions on physician wellness and resilience (Vanderbilt University Medical Center) **($)**  [United States]. |  |  |  |  |
|  | [**Code Lavender ® Program**](https://www.vocera.com/public/ein/Misc/Code-Lavender-Executive-Summary.pdf)  –This solution-focused toolkit outlines how Code Lavender® programs can be implemented to support the physical, emotional, and spiritual well-being of patients, families, and care team members. A Code Lavender Program is a formalized rapid response designed to support patients, families, physicians, nurses, and staff members in times of emotional distress  [United States]. |  |  |  |  |
|  | [**Caring for Healthcare Workers**](https://www.caringforhealthcareworkers.com/)  – A resource for Canadian healthcare organizations to assess and promote workplace psychological health and safety  [Canada]. |  |  |  |  |
|  | [**Caring Greatly Podcast**](https://www.vocera.com/podcast)  – A destination where healthcare leaders find stories and resources designed to help them to grow, lead, innovate, and rejuvenate  [United States]. |  |  |  |  |
|  | [**Human-centered leadership mastery model**](https://www.vocera.com/sites/default/files/2021-12/HCL.2206.report.202006.WEB-rev.pdf) – Based on in-person discussions and design sessions as well as interviews with more than 75 executives, thought leaders, and academics in healthcare and well-being, this report defines the heartset, mindset, and skillset that enable leaders to support themselves, lead their teams, and drive sustainable organizational excellence  [United States]. |  |  |  |  |
|  | [**Compassion Resilience Toolkit for Health and Human Services Leaders and Staff**](https://compassionresiliencetoolkit.org/healthcare/a-toolkit-for-healthcare/)  **–** This toolkit explores ways to maintain a compassionate presence in interactions with clients, their families and colleagues. The rationale for this work highlights the importance of compassion and resilience within the health care field and points to the many positive outcomes for clients, providers, and organizations as a whole  [United States]. |  |  |  |  |
|  | [**Holistic Transcendental Leadership Model**](C://Users/magda/Downloads/Barr%20and%20Nathenson%202022.pdf)  – A leadership model to enhance innovation and creativity while placing a novel emphasis on the physical, emotional, and spiritual well-being at the  individual, group, and organizational level  [United States]. |  |  |  |  |
|  | [**Creating a Just Culture Policy**](https://justculture.hqca.ca/creating-a-just-culture-policy/)  – A just culture policy is a high-level statement of the values and commitment of an organization to treat healthcare workers fairly when a patient is harmed or nearly harmed by healthcare delivery  [Canada, Alberta]. |  |  |  |  |
|  | **[Transforming Healthcare](https://www.mentalhealthcommission.ca/wp-content/uploads/drupal/2018-11/healthcare_crosswalk_eng.pdf)**  **[Organizations](https://www.mentalhealthcommission.ca/wp-content/uploads/drupal/2018-11/healthcare_crosswalk_eng.pdf)**  – This document is intended to assist healthcare leaders to understand synergies of the National Standard of Canada for Psychological Health and Safety in the Workplace (The Standard) and LEADS and to support them in implementing both frameworks within their organizations  [Canada]. |  |  |  |  |
|  | [**Reach for Resilience – Burnout Toolkit**](https://reach4resiliencend.com/wp-content/uploads/2022/01/Reach-for-Resilience-Burnout-Toolkit_.pdf)  **–** Strategies to prevent and combat burnout  [United States]. |  |  |  |  |
|  | [**Healthy Workplace Matters**](https://oasisdiscussions.ca/healthy-workplace-matters/)  **–** An initiative that brings together helpful information and resources in one convenient location to help employers and the whole dental team participate in creating a healthier and more respectful workplace (Canadian Dental Association, Canadian Dental Assistants Association and Canadian Dental Hygienist Association)  [Canada]. |  |  |  |  |
| **Addressing Burnout** | [**Rapid access to treatment and rehabilitation for NHS staff**](https://www.nhsemployers.org/articles/rapid-access-treatment-and-rehabilitation-nhs-staff)  – A guidance that provides information for NHS organisations looking to implement and/or review current rapid access processes  [United Kingdom]. |  | [**Violence and aggression incident investigation checklist**](https://www.commerce.wa.gov.au/sites/default/files/atoms/files/violence_and_aggression_health_care_form.pdf) – This checklist designed for employees, OSH Reps or supervisors to assist with investigation of incidents resulting from violence and aggression from clients/ residents or visitors  [Australia]. |  |  |
|  | [**Stress First Aid for Health Care Workers**](https://www.ptsd.va.gov/professional/treat/type/stress_first_aid.asp)  – A framework to improve recovery from stress reactions both in oneself and in coworkers; includes training materials and other resources  [United States]. |  |  |  |  |
|  | [**Workplace Well-being and Mental Health Toolkit**](https://workplaces.healthiertogether.ca/media/uploads/healthier%20toegther_wellbeingtoolkit_ahs.pdf)  – Evidence-based actionable strategies to help workplaces increase support for individual mental health in the workplace   [Canada, Alberta]. |  |  |  |  |
|  | [**Tackling Stress Management, Addiction, and Suicide Prevention in a Predoctoral Dental Curriculum**](https://static1.squarespace.com/static/566e2c0f4bf118e6b4540a14/t/56a1261b1c12101924f67b8b/1453401628028/1286.full.pdf)  – A module designed to address stress management and suicide prevention among health workers, particularly dentists. The pedagogies include standardized patients, invited guest lectures, in-class activities, video presentation, and self-reflections (developed by the University of British Columbia Faculty of Dentistry)  [Canada, British Columbia]. |  |  |  |  |
|  | [**Moral Stress Amongst Healthcare Workers During COVID-19: A Guide to Moral Injury**](https://www.moralinjuryguide.ca/Documents/Moral-Injury-Guide.pdf)  – A practical resource for healthcare workers and organizations to better understand the range of moral emotions arising from the COVID-19 pandemic and to develop organizational and individual strategies to mitigate risks of lasting harm  [Australia]. |  |  |  |  |
| **Supporting Burnout Recovery** | [**Sickness absence toolkit**](https://www.nhsemployers.org/publications/sickness-absence-toolkit)  – A simple guide for managers about sickness absence (National Health Service)  [United Kingdom]. |  |  |  |  |
|  | [**Workplace Strategies for Mental Health**](https://www.workplacestrategiesformentalhealth.com/resources/accommodation-strategies)  – Accommodation strategies for employees with mental health issues who are at work or returning to work  [Canada]. |  |  |  |  |
|  | [**Mental Health in the Workplace**](https://www.mentalhealthworks.ca/wp-content/uploads/2021/12/CMHA_Mental-Health-Works-Guidebook.pdf)  – This guide outlines some key skills managers should have when presented with an employee who has a mental health concern  [Canada, Ontario]. |  |  |  |  |

#
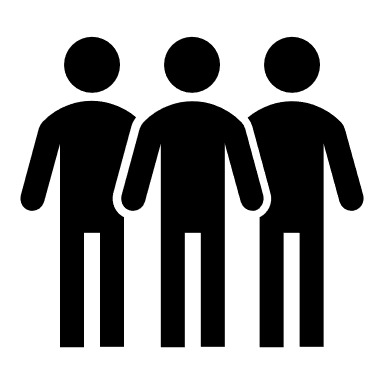
Team Level Tools, Toolkits & Interventions

* **Team** level toolkits and interventions focus on peer support, mentorship and leadership within a group setting.

| *For Gender/ Intersectional Informed | **Promoting Mental Health** | **Managing Workload** | **Handling Conflict, Bullying & Harassment** | **Addressing Discrimination** | **Reducing Stigma & Facilitating Disclosure** |
| --- | --- | --- | --- | --- | --- |
| **Preventing Burnout** | [**How to Implement a Peer Support Program During a Crisis**](https://gateway.on24.com/wcc/eh/3346741/lp/3545167/how-to-implement-a-peer-support-program-during-a-crisis)  – A webinar that covers the importance of peer support, the fundamentals for operationalizing a peer-support program in health systems and practices, and how peer support can potentially change organizational culture – especially during the COVID-19 pandemic  [United States]. | **Medical scribes** –  A strategy to increase clinic workflow efficiency and reduce physician burnout; relies on the incorporation of medical assistants into electronic health re cord (EHR) processes  [United States]  ([Gao et al., 2019](https://ascopubs.org/doi/full/10.1200/JOP.19.00307); [DeChant et al., 2019](https://www.sciencedirect.com/science/article/pii/S2542454819300876)). | [**Assaultive Staff Action Program (ASAP)**](https://pubmed.ncbi.nlm.nih.gov/32483767/) –  A voluntary, system-wide, peer-help, crisis intervention program to address the psychological sequalae in staff victims of  patient assaults  [United States; Canada, Ontario]. | [**Queer Mentorship Program**](https://canadianmidwives.org/becoming-a-midwife/#mentorship) – The Canadian Caucus of Queer and Trans Midwives hosts a queer mentorship program to support student midwives in Canada. Primarily this will work as an online/phone program with each mentor‑mentee pair deciding how and how often they will interact  [Canada]. |  |
|  | [**Battle Buddies**](https://hsc.unm.edu/echo/_docs/frr-covid/09.21.20_battle_buddy_program,_part_2_minnrap_salbott.pdf)  – A psychological resilience intervention founded on a peer support model; involves pairing individuals together based on their clinical areas of practice, responsibilities, experience, seniority, and life circumstances [United States]  (See also: [Albott et al., 2020](https://journals.lww.com/anesthesia-analgesia/Fulltext/2020/07000/Battle_Buddies__Rapid_Deployment_of_a.9.aspx)). | [**Flattened hierarchy model**](https://bmjopenquality.bmj.com/content/bmjqir/10/3/e001415.full.pdf)  – A weakening of traditional hierarchies in a hospital setting that permits the delegation of tasks ‘down’ to the most invested stakeholder rather than ‘up’ to the most responsible one  [United States]  (See also: [Green et al., 2017](https://www.bjoms.com/article/S0266-4356(17)30061-X/fulltext)). | [**Civility, Respect, Engagement in the Workforce (CREW)**](https://www.va.gov/ncod/crew.asp)  – A culture change initiative to improve the work climate through more civil and respectful interactions (National Center for Organization Development, United States Department of Veterans Affairs)  [United States]. | [**BIPOC Mentorship Program**](https://canadianmidwives.org/becoming-a-midwife/#mentorship)  **– A program launched by Toronto Metropolitan University MEP for self-identified students who are Black, Indigenous, or People of Colour (BIPOC). This program is an opportunity for BIPOC midwives to pair, share and care with the next generation of BIPOC students**  **[Canada].** |  |
|  | [**Health and wellbeing conversations**](https://www.nhsemployers.org/articles/health-and-wellbeing-conversations#:~:text=Using%20a%20tool%20like%20the,support%20for%20your%20own%20wellbeing.)  – Regular health and wellbeing focused conversations held by line managers/ supervisors with their staff  [United Kingdom]. |  |  | [**Health Equity Rounds (HER)**](https://www.mededportal.org/doi/epdf/10.15766/mep_2374-8265.10858)  – A unique interdisciplinary forum for practitioners across training levels to discuss and address the impact of structural racism and implicit bias on patient care  [United States]. |  |
|  | [**Peer Support Drop-In Available to PEI Physicians**](https://www.mspei.org/covid/physician-wellness/)  – Peer support available for physicians - hosted by MH professionals (psychiatrists, physicians, etc.) to discuss any challenges physicians are facing MSPEI’s Physician Health Program, provided through Doctors of BC, is now hosting free drop-in COVID-19 Physician Peer Support Sessions via Zoom  [Canada, Prince Edward Island]. |  |  |  |  |
|  | [**Faculty support calls as an intervention**](https://www.sciencedirect.com/science/article/pii/S0022347620305618?pes=vor)  – A program of voluntary and informal 1-hour group support video calls to help medical faculty address their challenges, listen to how they are coping, and describe lessons learned  [United States]. |  |  |  |  |
|  | [**Mentorship Program**](https://onlinelibrary.wiley.com/doi/10.1002/aet2.10354)  – A support for resident-students/fourth-year medical students (during their 4-week emergency medicine sub internship)  [United States]. |  |  |  |  |
|  | [**Voices of Duke Health**](https://sites.duke.edu/listening/)  – A listening booth and podcast project that invites Duke Health providers, staff, students, trainees, patients, and visitors to have one-on-one conversations about what is meaningful in your lives, work, and relationships  [United States]. |  |  |  |  |
|  | [**Team Huddles**](https://onlinelibrary.wiley.com/doi/10.1111/jep.13648)  – A structured, brief (5-15 min) routine (daily or multiple times a day), face-to-face communication of a team’s membership; has been shown to improve patient safety by enhancing teamwork creating standardized communication processes and providing a feeling of shared responsibility.  [International] |  |  |  |  |
|  | [**Women in Dentistry**](https://womensdentistry.com/)  – Work, Life, Balance Group of female dentists gathered together to chat about an array of issues relevant to women in dental profession (clinical aspects, social and family life) (***intersectionally focused***)  [Canada, Ontario]. |  |  |  |  |
|  | [**Schwartz rounds**](https://www.theschwartzcenter.org/programs/schwartz-rounds/) –  A standardized rounding program designed to openly and honestly discuss social and emotional issues that health workers face in caring for patients and families **($)**  [United States].  (See also: [Maben et al, 2021](https://bmchealthservres.biomedcentral.com/articles/10.1186/s12913-021-06483-4)). |  |  |  |  |
|  | **Recharge rooms/ communal spaces:**  [**Wobble room**](https://www.interiorhealth.ca/stories/wobble-rooms-help-health-care-workers-unwind-and-connect) – A physical space for health workers to unwind and connect  [Canada]  (See also: [Gurney et al., 2020](https://www.researchgate.net/profile/Lara-Gurney-2/publication/349450387_Why_Do_We_Need_Wobble_Rooms_during_COVID-19/links/60da78c8299bf1ea9ecb391d/Why-Do-We-Need-Wobble-Rooms-during-COVID-19.pdf)).  [**Staff well-being hub**](https://bmjopenquality.bmj.com/content/bmjqir/9/3/e001008.full.pdf) – A designated positive space for hospital staff to help them detox and recuperate during the COVID-19 pandemic  [United States].  [**The Bubble**](https://www.jpsmjournal.com/article/S0885-3924(20)30755-7/fulltext) – A program and a space for relaxation and support for hospital-based health workers  [France]. |  |  |  |  |
|  | **[Team Strategies and Tools to Enhance Performance and](https://www.ahrq.gov/teamstepps/index.html)**  **[Patient Safety (TeamSTEPPS)](https://www.ahrq.gov/teamstepps/index.html)**  - A validated curriculum for teaching interprofessional communication; used with health workers; focuses on five key principles (leadership, situation monitoring, mutual support, communication, and team structure) **($)**  [United States]  (See also: [Chen et al., 2019](https://pubmed.ncbi.nlm.nih.gov/31009273/); [Marrs et al., 2020](https://pubmed.ncbi.nlm.nih.gov/31759766/)). |  |  |  |  |
|  | [**Balint 2.0: A virtual Balint group for doctors around the world**](https://journals.sagepub.com/doi/abs/10.1177/0091217418765036)  – Balint groups have shown promise in addressing clinician burnout. However, their traditional format of in-person sessions limits their ability to meet the needs of clinicians practicing in locations without trained Balint leaders. The authors of this scholarly publication report on a pilot of an international, internet-based Balint group in collaboration between the World Organization of Family Doctors regional Young Doctors Movements and the [International Balint Federation](https://www.balintinternational.com/) (See also: [Huang et al., 2019](https://www.ncbi.nlm.nih.gov/pmc/articles/PMC7026367/#:~:text=A%20randomized%20controlled%20trial%20design%20was%20used%20to,and%20modified%20by%20Tongji%20Hospital%20of%20Tongji%20University.)).  [International] |  |  |  |  |
|  | [**Death Cafés for prevention of burnout**](https://trialsjournal.biomedcentral.com/articles/10.1186/s13063-020-04929-4)  – Informal discussions focusing on death, dying, loss, grief, and illness. They allow for reflection on distressing events and offer community and collaboration among hospital employees outside of work. With Covid-19 limiting social interactions and overloading of ICUs worldwide, their virtual administration provides an innovative strategy to mitigate burnout  [United States]  (See also: [Hammer et al., 2021](https://mh.bmj.com/content/47/1/2)). |  |  |  |  |
|  | [**Escape room as a team building intervention in interprofessional healthcare teams**](https://commons.erau.edu/cgi/viewcontent.cgi?article=2692&context=publication)  – A novel approach to advance team cohesion in the healthcare setting  [United States]  (See also: [Guckian et al., 2020](https://www.ncbi.nlm.nih.gov/pmc/articles/PMC7296573/)). |  |  |  |  |
|  | [**Line Manager Competency Indicator Tool**](https://www.hse.gov.uk/stress/mcit.htm)  – Allows managers to assess whether they currently have the behaviors identified as effective for preventing and reducing stress at work  [United Kingdom]. |  |  |  |  |
|  | [**#2022 Healthcare Workforce Rescue Package**](https://www.allinforhealthcare.org/issues/2022-healthcare-workforce-rescue-package)  – Evidence-based actions that can support healthcare team member well-being. The first-of-its-kind list prioritizes five critical actions leaders can take now to safeguard the emotional and psychological needs of healthcare workers (published by a group of healthcare experts in collaboration with the CEO Coalition and the National Academy of Medicine)  [United States]. |  |  |  |  |
|  | [**Well-being debriefings for health care workers: An Evidence-based method for improving well-being**](https://www.capc.org/documents/download/929/) – A facilitator training manual to lead informal, peer-facilitated, small-group meetings where health workers have an opportunity to give voice to the difficult nature of their work and discuss issues that negatively affect resiliency  [Australia]. |  |  |  |  |
| **Addressing Burnout** | [**Reflective debriefing**](https://www.tandfonline.com/doi/abs/10.1080/15524256.2018.1437588?journalCode=wswe20)  – A protocol for regular case study/moral distress debriefings with ICU nursing staff, including an educational component in moral distress, moral efficacy, and common end-of-life issues  on the ICU (social work intervention)  [United States]. |  |  |  | [**Trauma risk management (TRiM)**](https://www.proquest.com/docview/1898070100?accountid=14701&parentSessionId=3ITJNtbyH3MTn3ay7L4che3bed0H7WzeYU2DETwNSeI%3D&pq-origsite=primo&forcedol=true)  – A structured peer-support process/system of risk assessment and post trauma support aimed ***to reduce stigma*** associated with help seeking; it relies on training volunteer nonmedical staff from within the organization to be TriM practitioners; see p. 123 for an example of a “typical TRiM managed incident”  [United Kingdom]. |
|  | [**Meaning-Centered Team-Level Intervention**](https://www.mdpi.com/1660-4601/19/13/7801/htm)  **–** This article promotes the use Meaning-Centered Psychotherapy (MCP) (a brief, evidence-based intervention designed for patients with advanced cancer); it describes the principles underlying MCP and how it might be adapted and applied to ameliorate burnout among health care workers. |  |  |  | [**Beyond Silence Champion Training**](https://www.beyondsilence.ca/training/)  – An evidence-based workplace mental health training course designed specifically for healthcare workers and led by trained healthcare employees with mental health experience. It combines information sharing and skill development with strategies for ***stigma reduction***  [Canada, Ontario]. |
| **Supporting Burnout Recovery** | [**Sample script for approaching distressed physicians**](https://edhub.ama-assn.org/steps-forward/module/2702599)  – This sample script from the American Medical Association provides guidance on how to approach a colleague who seems distressed along with some talking points to help prepare and support physicians offering peer support  [United States]. |  | [**Follow-Up Counselling**](https://pubmed.ncbi.nlm.nih.gov/32369903/)  – An important step to help minimize the adverse impact of exposure to aggression from patients on nurses' mental health (***multileveled***). |  | [**The Working Mind: Workplace Mental Health & Wellness for Oral Health Care Professionals**](https://nsdental.org/agm2022/ce-opportunities/) – A course that covers various topics related to mental health, including how to communicate with employees about mental health illness in a workplace context and workplace accommodations and return to work and how to how ***to reduce associated stigma*** (Nova Scotia Dental Association)  [Canada]. |
|  | **[Returning to the Workplace --](https://www.wechu.org/sites/default/files/edit-resource/em-wtw-committee-resources/fseap-2021-return-workplace-resiliency-toolkit-managers.pdf)**  **[Resiliency Toolkit for Managers](https://www.wechu.org/sites/default/files/edit-resource/em-wtw-committee-resources/fseap-2021-return-workplace-resiliency-toolkit-managers.pdf)**  – Provides information, strategies, tips, and resources to guide managers and people leaders in their work to support employee and team well-being and resiliency  [Canada]. |  |  |  |  |

#
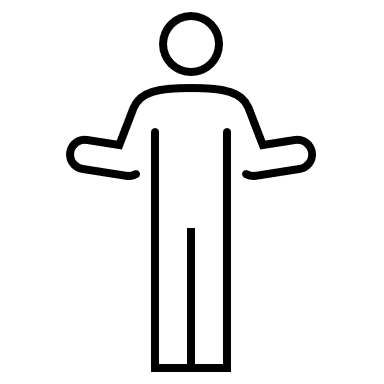
Individual Level Tools, Toolkits & Interventions

* **Individual** level toolkits must focus on worker empowerment, changes in work culture or aimed at leadership within a division. We are not including single interventions that place the onus on the individual to ‘fix’ themselves.

| *For Gender/ Intersectional Informed | **Promoting Mental Health** | **Managing Workload** | **Handling Conflict, Bullying & Harassment** | **Confronting**  **Discrimination** | **Reducing Stigma & Facilitating Disclosure** |
| --- | --- | --- | --- | --- | --- |
| **Preventing Burnout** | [**Vitalk app**](https://vitalk.health/)  – A virtual health assistant, designed to interact with health workers and screen them for mental health disorders—anxiety, burnout, and depression and refers health workers needing more complex resources to a human service provider  [Brazil]. | [**Psychosocial Factors: Workload Management**](https://www.csps-efpc.gc.ca/video/workload-management-eng.aspx)  – A short video on workload management  [Canada]. | [**Conflict Resolution Process and Bullying**](https://mentalhealth.csmls.org/i-am-an-employee/#sd)  **–** This webpage provides information on how to deal with conflict and bullying as an employee (Canadian Society for Medical Laboratory Science)  [Canada]. | [**Virtual Simulations to Confront Racism and Bias in Health Professions Education**](https://www.sciencedirect.com/science/article/abs/pii/S1876139922000305) – This educational series was developed in consultation with experts to utilize best practices in racial and social justice simulation. | [**The Working Mind Virtual**](https://mentalhealthcommission.ca/training/twm/the-working-mind-virtual/) –  An evidence-based program designed to promote mental health and reduce the stigma around mental illness in the workplace  [Canada]. |
|  | [**Drop-in mindfulness sessions for healthcare providers**](https://www.pause4providers.com/)  – 30-minute online mindfulness sessions guided by Canadian healthcare providers to pause and make space to cultivate well-being and self-compassion. Sessions are open to healthcare workers, staff and professionals at no cost  [Canada]. | [**Setting Boundaries as a Health Care Worker**](https://mhanational.org/setting-boundaries-health-care-worker) (Mental Health America)  [United States]. |  | [**Mental Health at Work – Being anti-racist in the workplace**](https://www.mentalhealthatwork.org.uk/toolkit/being-anti-racist-in-the-workplace/) – Videos and articles on anti-racism in the workplace  [United Kingdom]. | [**Stigma**](https://mentalhealth.csmls.org/i-am-an-employee/#sd) – This webpage provides information on how to deal with stigma as an employee (Medical Laboratory Science)  [Canada]. |
|  | [**Psychological First Aid for Frontline Health Care Providers During COVID-19**](https://policy.nshealth.ca/Site_Published/covid19/document_render.aspx?documentRender.IdType=6&documentRender.GenericField=&documentRender.Id=79548)  **–** A resource/ handbook for frontline health professionals to work through to improve well being during Covid-19 pandemic  [Canada, Nova Scotia]. |  |  | [**Do I Belong Here?**](https://www.mededportal.org/doi/10.15766/mep_2374-8265.11166) **–** A workshop that aims to discuss the impact of Imposter Syndrome and develop strategies to confront it at the individual, peer, and institutional levels (see Appendices for workshop resources)  [United States]. |  |
|  | [**Expressive arts**](https://onlinelibrary.wiley.com/doi/10.1111/jan.14043)  – Used to address psychosocial stress in health workers; provide a creative means for reducing health worker stress to remain well and able to provide high quality care to patients.  See also: [**Creative Arts Therapy as a potential intervention to prevent burnout and build resilience in health care professionals**](https://pubmed.ncbi.nlm.nih.gov/32526006/). |  |  |  |  |
|  | [**Anticipate-Plan-Deter (APD) model**](https://nchph.org/5331-2/) – A strategy for mitigating psychological consequences for health workers who are responding to disaster  (See also: [Albott et al., 2020](https://www.ncbi.nlm.nih.gov/pmc/articles/PMC7199769/)). [**Resilience Roadmap**](https://www.nebraskamed.com/sites/default/files/documents/covid-19/pandemic-resilience-roadmap.pdf) – A handout modeled after Anticipate, Plan, Deter. |  |  |  |  |
|  | [**Mindarma (previously known as RAW mindfulness)**](https://mindarma.com/home/the-lowdown/#:~:text=Mindarma%20is%20designed%20to%20help%20workers%20become%20more,turnover%2C%20and%20workers%E2%80%99%20compensation.%20Ongoing%20learning%20and%20engagement)  – An online evidence-based intervention designed to increase resilience in high-risk workers (combines principles from mindfulness, CBT, acceptance and commitment therapy, and self-compassion approaches) **($)**  (see also: [Hooper et al., 2021](https://pubmed.ncbi.nlm.nih.gov/34020974/))  [Australia]. |  |  |  |  |
|  | [**Careforcaregivers**](https://www.careforcaregivers.ca/)  – This website provides a diverse collection of quick and easy resources to support the mental health of health workers  [Canada, British Columbia]. |  |  |  |  |
|  | [**Strengthening Mental Health and Wellness During COVID-19**](https://www.oha.com/news/strengthening-mental-health-and-wellness-during-covid-19)  – Easily accessible mental health resources for Ontario’s health care workers during COVID-19 (Ontario Hospital Association)  [Canada, Ontario]. |  |  |  |  |
|  | [**Animal-assisted support program for healthcare employees**](https://bmchealthservres.biomedcentral.com/articles/10.1186/s12913-020-05586-8)  – Research findings suggested that employees were highly satisfied with the program, noticed an improved clinic atmosphere, and experienced a reduction in stress and boost in mood  [United States]. |  |  |  |  |
|  | [**Guideline on Managing Personal and Practice Health**](https://www.cmo.on.ca/wp-content/uploads/2018/06/Guideline-on-Managing-Personal-Practice-Health-final.pdf)  – Provides information on supports and resources for midwives who are experiencing mental health and addiction issues (Association of Ontario Midwives)  [Canada, Ontario]. |  |  |  |  |
|  | [**ECHO - Coping with COVID**](https://www.nbasw-atsnb.ca/news-and-events/continuing-education/echo-coping-with-covid-19/)  – This virtual education and capacity-building program is designed for health care providers and residents working in the health care system during the pandemic to share and learn about ways to build resilience and wellness through didactic lectures and case-based discussions (New Brunswick Association of Social Workers)  [Canada, New Brunswick]. |  |  |  |  |
|  | [**RESPITE: Resilience in the Era of Sustainable Physicians: An International Training Endeavour**](https://respite.machealth.ca/about.html)  – An international, multisite, e-teaching endeavour currently offering an optional resilience curriculum; the online training program is available during each year of residency training to all physicians in training (residents and fellows); the curriculum includes the online module, reading material, and a post-module survey  [International]. |  |  |  |  |
|  | [**Pause, Reset and Nourish Toolkit (PRN)**](https://www.hamiltonhealthsciences.ca/covid19/staff-physician/hhs-resources/resilience-support-toolkit/)  – Strategies to help build connection to each other, ourselves and what is truly important in our lives (Hamilton Health Services)  [Canada, Ontario]. |  |  |  |  |
|  | [**Clinician Well-Being Knowledge Hub**](https://nam.edu/clinicianwellbeing/)  **–** The National Academy of Medicine’s Clinician Well-Being Knowledge Hub is a repository providing clinicians with resources relating to well-being and burnout, the impact that it can have on patients and their families, and the practices organizations can implement to promote well-being. The resources include toolkits, blog posts, reports, research articles and briefs  [United States]. |  |  |  |  |
| **Addressing Burnout** | [**Moral distress and COVID-19: how to recognize and cope with it**](https://boldly.cma.ca/blog/moral-distress-and-covid-19)  – This webpage highlights the Canadian Medical Protective Association’s podcast with psychiatrist Dr. Caroline Gérin-Lajoie where she describes moral distress, how to recognize when someone is experiencing moral distress and how to reduce its impact  [Canada]. | [**BounceBack**](https://bouncebackontario.ca/)  – A free skill-building program designed to help adults and youth 15+ manage low mood, mild to moderate depression and anxiety, stress or worry. Delivered over the phone with a coach and through online videos, it gives access to tools that support mental wellness (Canadian Mental Health Association)  [Canada, Ontario]. |  |  | [**Words Matter: An anti-bias Workshop for Health Care Professionals**](https://www.mededportal.org/doi/10.15766/mep_2374-8265.11115)  – An interactive workshop to teach health care professionals a framework to identify and replace stigmatizing language in clinical practice (see Appendices for resources)  [United States]. |
|  | [**Introduction to Vicarious Trauma for Frontline Workers: A webinar**](https://cmhanb.ca/events/introduction-to-vicarious-trauma-for-frontline-workers/)  – This series helps frontline workers in developing an awareness and connection between the impacts of one’s own experiences, how to recognize signs leading to vicarious trauma, and learning strategies to help address vicarious trauma (Canadian Mental Health Association) [Canada]. |  |  |  |  |
| **Supporting Burnout Recovery** | [**Returning to a shared workspace: A psychological toolkit for transitioning to a new normal**](https://ontario.cmha.ca/wp-content/uploads/2019/12/CMHA_ReturnToWorkplace-Toolkit_EN_jan-2022_FINAL.pdf)  – A guide to support the mental health of individuals as they plan safe transitions back into their employer’s shared workspaces, and designed to help employers as they develop policies and procedures for supporting staff returning to a shared workspace (***multileveled***) (Mental Health Commission of Canada)  [Canada]. |  |  |  |  |

Sources

1. World Health Organization. Burn-out an “occupational phenomenon”: International Classification of Diseases [Internet]. 2019 [cited 2022 Nov 11]. Available from: <https://www.who.int/news/item/28-05-2019-burn-out-an-occupational-phenomenon-international-classification-of-diseases> [↑](#endnote-ref-1)
2. Ahola K., Toppinen-Tanner S., Seppänen J. Interventions to alleviate burnout symptoms and to support return to work among employees with burnout: Systematic review and meta-analysis. Burnout Research. 2017 Mar; 4:1–11. [↑](#endnote-ref-2)
3. Ibid [↑](#endnote-ref-3)
4. Perski O., Grossi G., Perski A., Niemi M. A systematic review and meta-analysis of tertiary interventions in clinical burnout. Scand J Psychol. 2017 Dec; 58(6): 551-61. [↑](#endnote-ref-4)
5. Ahola K, Toppinen-Tanner S, Seppänen J. Interventions to alleviate burnout symptoms and to support return to work among employees with burnout: Systematic review and meta-analysis. Burnout Research. 2017 Mar;4:1–11. [↑](#endnote-ref-5)
6. Canadian Mental Health Association Ontario. Mental Health in the Workplace: An Accommodation Guide for Managers and Staff [Internet]. 2017. Available from: <https://ontario.cmha.ca/wp-content/uploads/2018/10/CMHA_Mental-Health-Works-Guidebook-8.5-x11r.pdf> [↑](#endnote-ref-6)
7. WorkSafeBC. Bullying & harassment [Internet]. 2022 [cited 2022 Nov 11]. Available from: <https://www.worksafebc.com/en/health-safety/hazards-exposures/bullying-harassment> [↑](#endnote-ref-7)
8. Canadian Labour Relations. Workplace Discrimination Is a Common Problem [Internet]. 2021 [cited 2022 Nov 11]. Available from: <https://www.canadianlabourrelations.com/workplace-discrimination.html> [↑](#endnote-ref-8)
9. Link BG, Phelan JC. Conceptualizing Stigma. Annual Review of Sociology. 2001;27(1):363–85. [↑](#endnote-ref-9)
10. Canadian Mental Health Association. Mental Illnesses in the Workplace [Internet]. 2022 [cited 2022 Nov 11]. Available from: <https://ontario.cmha.ca/documents/mental-illnesses-in-the-workplace> [↑](#endnote-ref-10)
